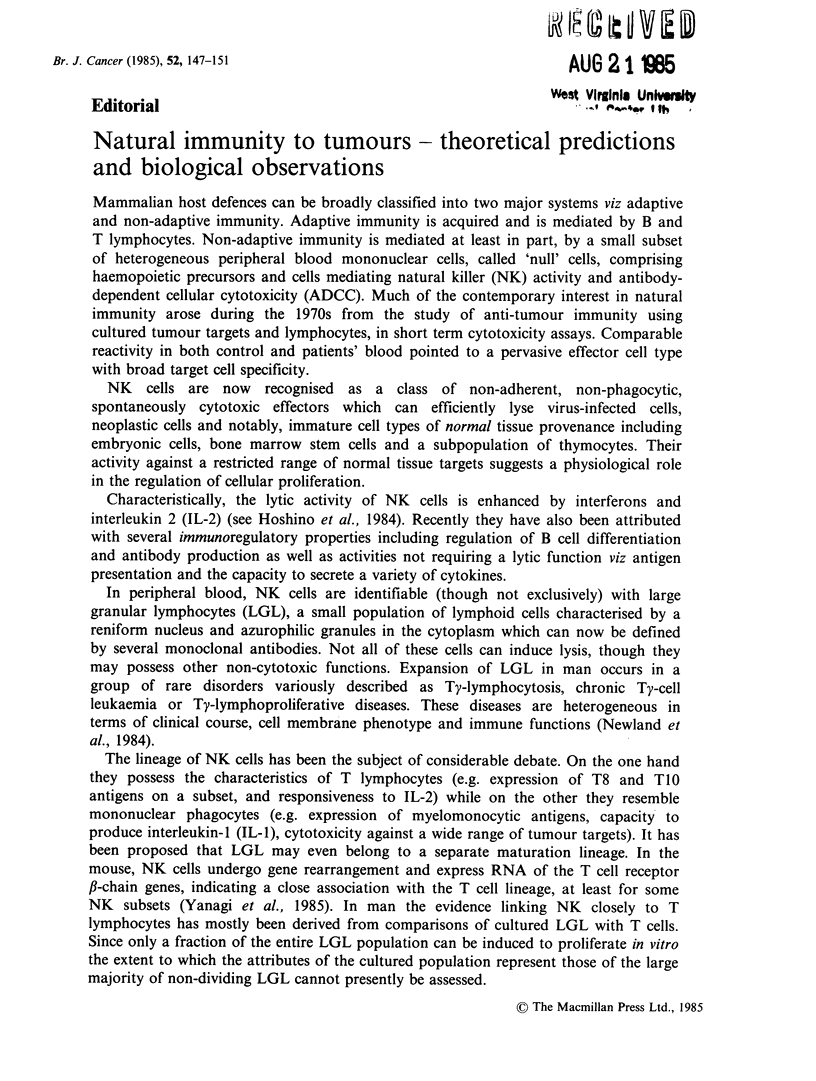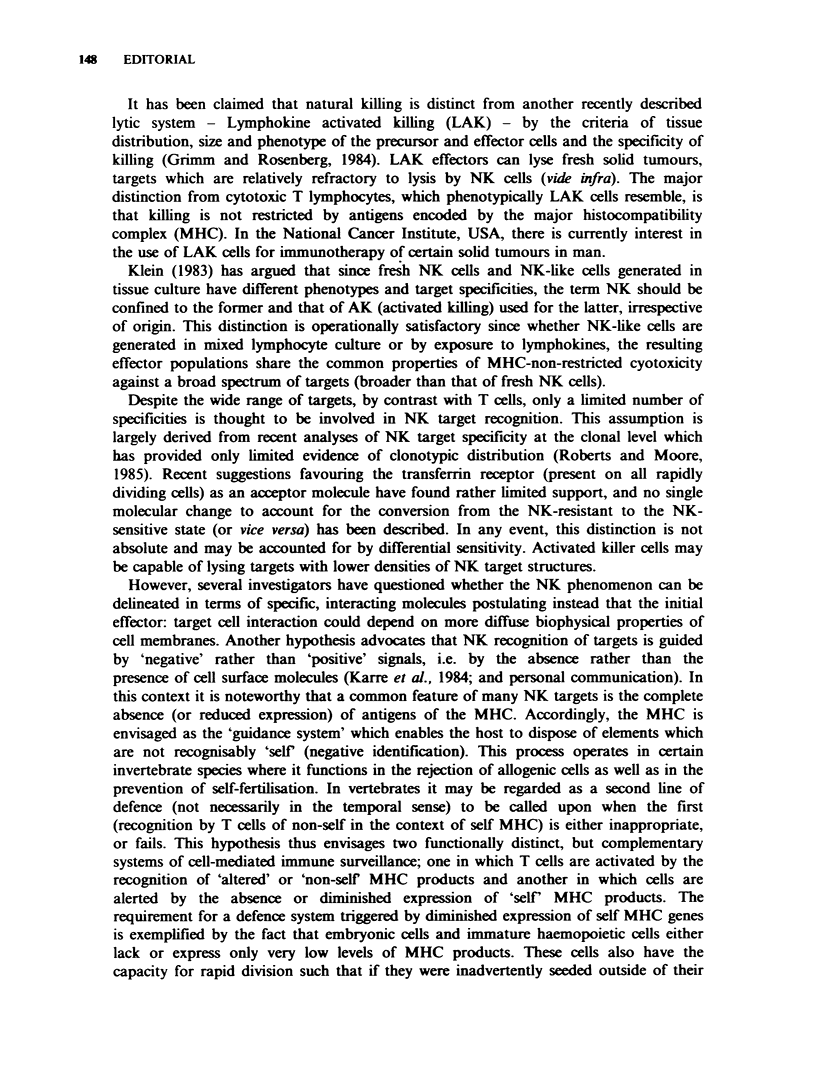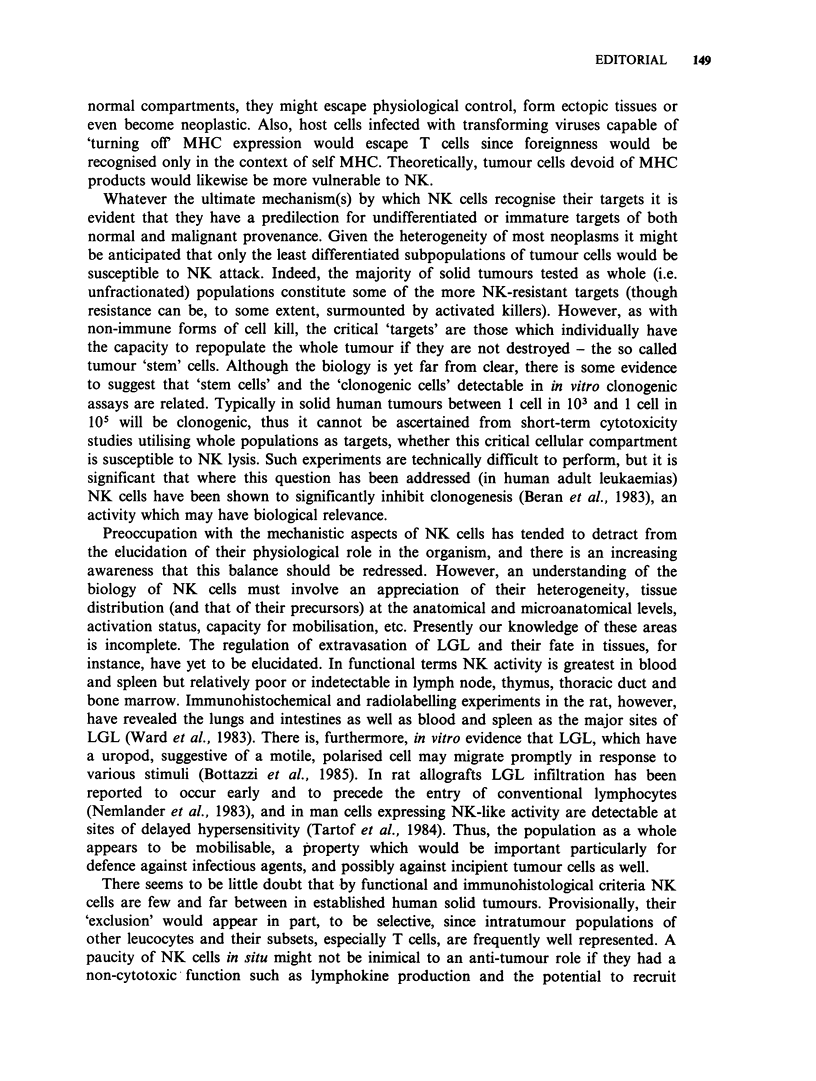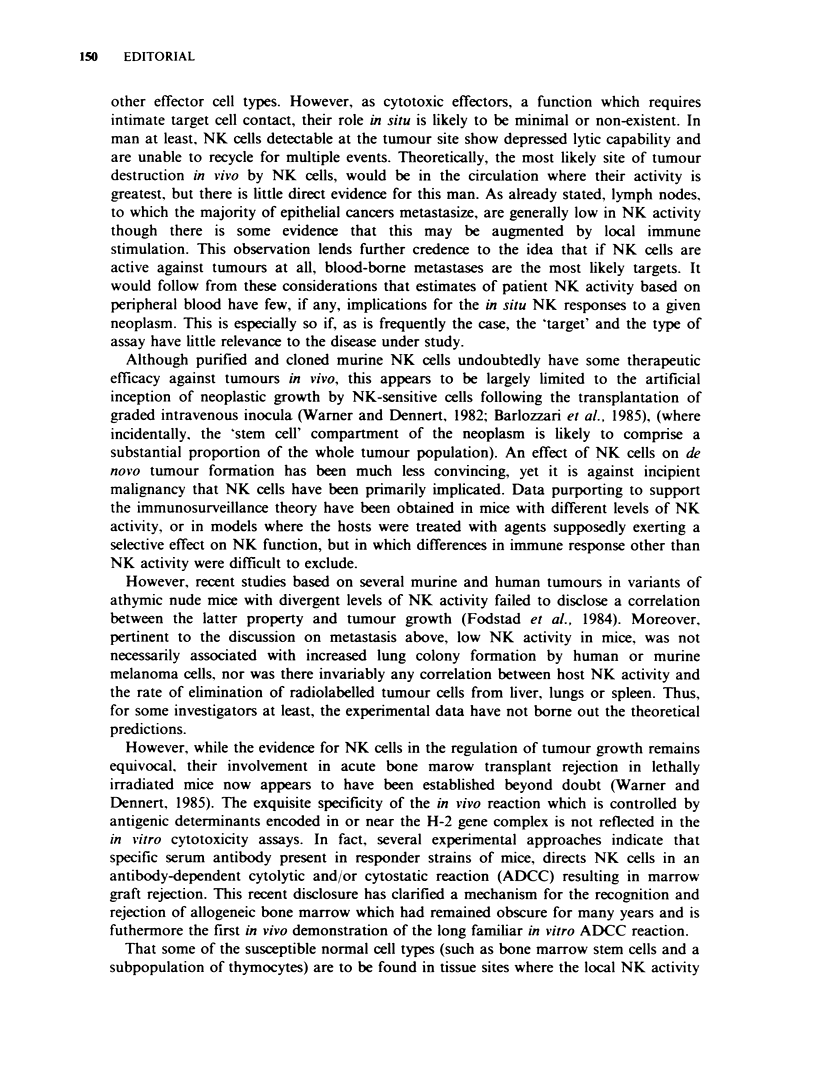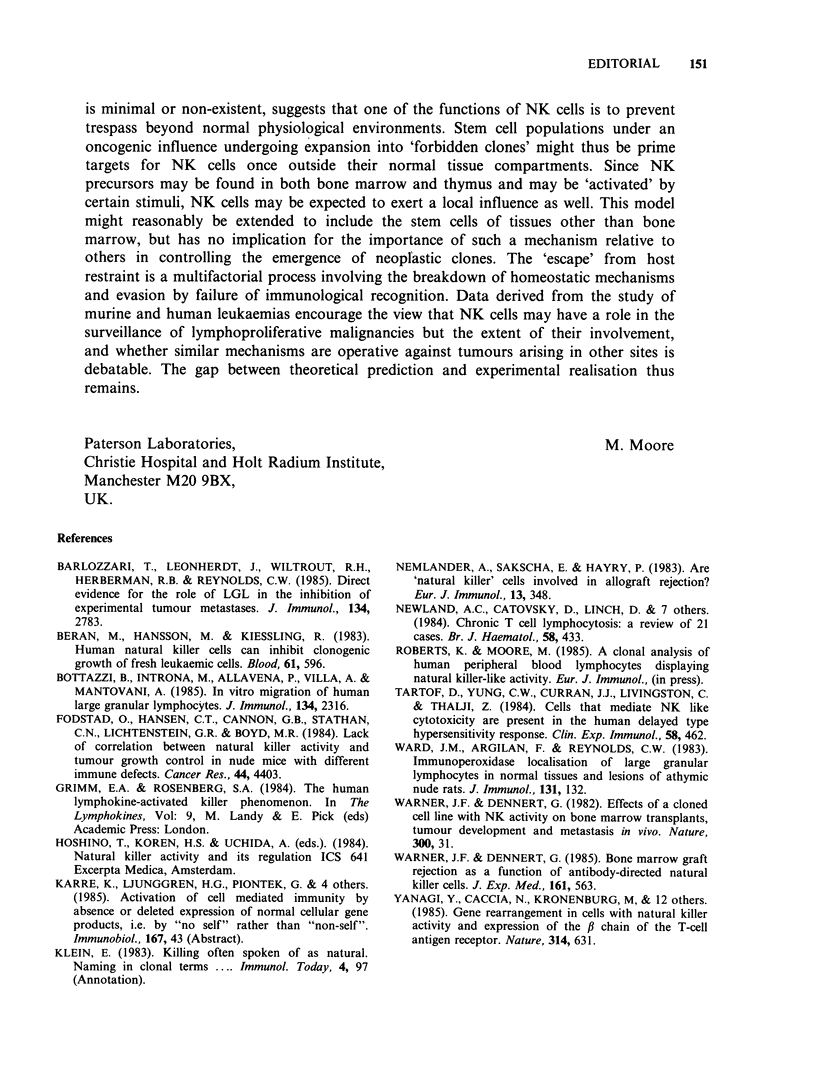# Natural immunity to tumours--theoretical predictions and biological observations.

**DOI:** 10.1038/bjc.1985.170

**Published:** 1985-08

**Authors:** M. Moore


*Br. J. Cancer* (1985), **52**, 147–151

**Editorial**

### Natural immunity to tumours – theoretical predictions and biological observations

Mammalian host defences can be broadly classified into two major systems *viz* adaptive and non-adaptive immunity. Adaptive immunity is acquired and is mediated by B and T lymphocytes. Non-adaptive immunity is mediated at least in part, by a small subset of heterogeneous peripheral blood mononuclear cells, called 'null' cells, comprising haemopoietic precursors and cells mediating natural killer (NK) activity and antibody-dependent cellular cytotoxicity (ADCC). Much of the contemporary interest in natural immunity arose during the 1970s from the study of anti-tumour immunity using cultured tumour targets and lymphocytes, in short term cytotoxicity assays. Comparable reactivity in both control and patients' blood pointed to a pervasive effector cell type with broad target cell specificity.

NK cells are now recognised as a class of non-adherent, non-phagocytic, spontaneously cytotoxic effectors which can efficiently lyse virus-infected cells, neoplastic cells and notably, immature cell types of *normal* tissue provenance including embryonic cells, bone marrow stem cells and a subpopulation of thymocytes. Their activity against a restricted range of normal tissue targets suggests a physiological role in the regulation of cellular proliferation.

Characteristically, the lytic activity of NK cells is enhanced by interferons and interleukin 2 (IL-2) (see Hoshino *et al.*, 1984). Recently they have also been attributed with several *immuno*regulatory properties including regulation of B cell differentiation and antibody production as well as activities not requiring a lytic function *viz* antigen presentation and the capacity to secrete a variety of cytokines.

In peripheral blood, NK cells are identifiable (though not exclusively) with large granular lymphocytes (LGL), a small population of lymphoid cells characterised by a reniform nucleus and azurophilic granules in the cytoplasm which can now be defined by several monoclonal antibodies. Not all of these cells can induce lysis, though they may possess other non-cytotoxic functions. Expansion of LGL in man occurs in a group of rare disorders variously described as T$\gamma$-lymphocytosis, chronic T$\gamma$-cell leukaemia or T$\gamma$-lymphoproliferative diseases. These diseases are heterogeneous in terms of clinical course, cell membrane phenotype and immune functions (Newland *et al.*, 1984).

The lineage of NK cells has been the subject of considerable debate. On the one hand they possess the characteristics of T lymphocytes (e.g. expression of T8 and T10 antigens on a subset, and responsiveness to IL-2) while on the other they resemble mononuclear phagocytes (e.g. expression of myelomonocytic antigens, capacity to produce interleukin-1 (IL-1), cytotoxicity against a wide range of tumour targets). It has been proposed that LGL may even belong to a separate maturation lineage. In the mouse, NK cells undergo gene rearrangement and express RNA of the T cell receptor $\beta$-chain genes, indicating a close association with the T cell lineage, at least for some NK subsets (Yanagi *et al.*, 1985). In man the evidence linking NK closely to T lymphocytes has mostly been derived from comparisons of cultured LGL with T cells. Since only a fraction of the entire LGL population can be induced to proliferate *in vitro* the extent to which the attributes of the cultured population represent those of the large majority of non-dividing LGL cannot presently be assessed.

© The Macmillan Press Ltd., 1985

It has been claimed that natural killing is distinct from another recently described lytic system – Lymphokine activated killing (LAK) – by the criteria of tissue distribution, size and phenotype of the precursor and effector cells and the specificity of killing (Grimm and Rosenberg, 1984). LAK effectors can lyse fresh solid tumours, targets which are relatively refractory to lysis by NK cells (*vide infra*). The major distinction from cytotoxic T lymphocytes, which phenotypically LAK cells resemble, is that killing is not restricted by antigens encoded by the major histocompatibility complex (MHC). In the National Cancer Institute, USA, there is currently interest in the use of LAK cells for immunotherapy of certain solid tumours in man.

Klein (1983) has argued that since fresh NK cells and NK-like cells generated in tissue culture have different phenotypes and target specificities, the term NK should be confined to the former and that of AK (activated killing) used for the latter, irrespective of origin. This distinction is operationally satisfactory since whether NK-like cells are generated in mixed lymphocyte culture or by exposure to lymphokines, the resulting effector populations share the common properties of MHC-non-restricted cyotoxicity against a broad spectrum of targets (broader than that of fresh NK cells).

Despite the wide range of targets, by contrast with T cells, only a limited number of specificities is thought to be involved in NK target recognition. This assumption is largely derived from recent analyses of NK target specificity at the clonal level which has provided only limited evidence of clonotypic distribution (Roberts and Moore, 1985). Recent suggestions favouring the transferrin receptor (present on all rapidly dividing cells) as an acceptor molecule have found rather limited support, and no single molecular change to account for the conversion from the NK-resistant to the NK-sensitive state (or *vice versa*) has been described. In any event, this distinction is not absolute and may be accounted for by differential sensitivity. Activated killer cells may be capable of lysing targets with lower densities of NK target structures.

However, several investigators have questioned whether the NK phenomenon can be delineated in terms of specific, interacting molecules postulating instead that the initial effector: target cell interaction could depend on more diffuse biophysical properties of cell membranes. Another hypothesis advocates that NK recognition of targets is guided by 'negative' rather than 'positive' signals, i.e. by the absence rather than the presence of cell surface molecules (Karre *et al.*, 1984; and personal communication). In this context it is noteworthy that a common feature of many NK targets is the complete absence (or reduced expression) of antigens of the MHC. Accordingly, the MHC is envisaged as the 'guidance system' which enables the host to dispose of elements which are not recognisably 'self' (negative identification). This process operates in certain invertebrate species where it functions in the rejection of allogenic cells as well as in the prevention of self-fertilisation. In vertebrates it may be regarded as a second line of defence (not necessarily in the temporal sense) to be called upon when the first (recognition by T cells of non-self in the context of self MHC) is either inappropriate, or fails. This hypothesis thus envisages two functionally distinct, but complementary systems of cell-mediated immune surveillance; one in which T cells are activated by the recognition of 'altered' or 'non-self' MHC products and another in which cells are alerted by the absence or diminished expression of 'self' MHC products. The requirement for a defence system triggered by diminished expression of self MHC genes is exemplified by the fact that embryonic cells and immature haemopoietic cells either lack or express only very low levels of MHC products. These cells also have the capacity for rapid division such that if they were inadvertently seeded outside of their normal compartments, they might escape physiological control, form ectopic tissues or even become neoplastic. Also, host cells infected with transforming viruses capable of 'turning off' MHC expression would escape T cells since foreignness would be recognised only in the context of self MHC. Theoretically, tumour cells devoid of MHC products would likewise be more vulnerable to NK.

Whatever the ultimate mechanism(s) by which NK cells recognise their targets it is evident that they have a predilection for undifferentiated or immature targets of both normal and malignant provenance. Given the heterogeneity of most neoplasms it might be anticipated that only the least differentiated subpopulations of tumour cells would be susceptible to NK attack. Indeed, the majority of solid tumours tested as whole (i.e. unfractionated) populations constitute some of the more NK-resistant targets (though resistance can be, to some extent, surmounted by activated killers). However, as with non-immune forms of cell kill, the critical 'targets' are those which individually have the capacity to repopulate the whole tumour if they are not destroyed – the so called tumour 'stem' cells. Although the biology is yet far from clear, there is some evidence to suggest that 'stem cells' and the 'clonogenic cells' detectable in *in vitro* clonogenic assays are related. Typically in solid human tumours between 1 cell in $10^3$ and 1 cell in $10^5$ will be clonogenic, thus it cannot be ascertained from short-term cytotoxicity studies utilising whole populations as targets, whether this critical cellular compartment is susceptible to NK lysis. Such experiments are technically difficult to perform, but it is significant that where this question has been addressed (in human adult leukaemias) NK cells have been shown to significantly inhibit clonogenesis (Beran *et al.*, 1983), an activity which may have biological relevance.

Preoccupation with the mechanistic aspects of NK cells has tended to detract from the elucidation of their physiological role in the organism, and there is an increasing awareness that this balance should be redressed. However, an understanding of the biology of NK cells must involve an appreciation of their heterogeneity, tissue distribution (and that of their precursors) at the anatomical and microanatomical levels, activation status, capacity for mobilisation, etc. Presently our knowledge of these areas is incomplete. The regulation of extravasation of LGL and their fate in tissues, for instance, have yet to be elucidated. In functional terms NK activity is greatest in blood and spleen but relatively poor or indetectable in lymph node, thymus, thoracic duct and bone marrow. Immunohistochemical and radiolabelling experiments in the rat, however, have revealed the lungs and intestines as well as blood and spleen as the major sites of LGL (Ward *et al.*, 1983). There is, furthermore, *in vitro* evidence that LGL, which have a uropod, suggestive of a motile, polarised cell may migrate promptly in response to various stimuli (Bottazzi *et al.*, 1985). In rat allografts LGL infiltration has been reported to occur early and to precede the entry of conventional lymphocytes (Nemlander *et al.*, 1983), and in man cells expressing NK-like activity are detectable at sites of delayed hypersensitivity (Tartof *et al.*, 1984). Thus, the population as a whole appears to be mobilisable, a property which would be important particularly for defence against infectious agents, and possibly against incipient tumour cells as well.

There seems to be little doubt that by functional and immunohistological criteria NK cells are few and far between in established human solid tumours. Provisionally, their 'exclusion' would appear in part, to be selective, since intratumour populations of other leucocytes and their subsets, especially T cells, are frequently well represented. A paucity of NK cells *in situ* might not be inimical to an anti-tumour role if they had a non-cytotoxic function such as lymphokine production and the potential to recruit other effector cell types. However, as cytotoxic effectors, a function which requires intimate target cell contact, their role *in situ* is likely to be minimal or non-existent. In man at least, NK cells detectable at the tumour site show depressed lytic capability and are unable to recycle for multiple events. Theoretically, the most likely site of tumour destruction *in vivo* by NK cells, would be in the circulation where their activity is greatest, but there is little direct evidence for this man. As already stated, lymph nodes, to which the majority of epithelial cancers metastasize, are generally low in NK activity though there is some evidence that this may be augmented by local immune stimulation. This observation lends further credence to the idea that if NK cells are active against tumours at all, blood-borne metastases are the most likely targets. It would follow from these considerations that estimates of patient NK activity based on peripheral blood have few, if any, implications for the *in situ* NK responses to a given neoplasm. This is especially so if, as is frequently the case, the 'target' and the type of assay have little relevance to the disease under study.

Although purified and cloned murine NK cells undoubtedly have some therapeutic efficacy against tumours *in vivo*, this appears to be largely limited to the artificial inception of neoplastic growth by NK-sensitive cells following the transplantation of graded intravenous inocula (Warner and Dennert, 1982; Barlozzari *et al.*, 1985), (where incidentally, the 'stem cell' compartment of the neoplasm is likely to comprise a substantial proportion of the whole tumour population). An effect of NK cells on *de novo* tumour formation has been much less convincing, yet it is against incipient malignancy that NK cells have been primarily implicated. Data purporting to support the immunosurveillance theory have been obtained in mice with different levels of NK activity, or in models where the hosts were treated with agents supposedly exerting a selective effect on NK function, but in which differences in immune response other than NK activity were difficult to exclude.

However, recent studies based on several murine and human tumours in variants of athymic nude mice with divergent levels of NK activity failed to disclose a correlation between the latter property and tumour growth (Fodstad *et al.*, 1984). Moreover, pertinent to the discussion on metastasis above, low NK activity in mice, was not necessarily associated with increased lung colony formation by human or murine melanoma cells, nor was there invariably any correlation between host NK activity and the rate of elimination of radiolabelled tumour cells from liver, lungs or spleen. Thus, for some investigators at least, the experimental data have not borne out the theoretical predictions.

However, while the evidence for NK cells in the regulation of tumour growth remains equivocal, their involvement in acute bone marow transplant rejection in lethally irradiated mice now appears to have been established beyond doubt (Warner and Dennert, 1985). The exquisite specificity of the *in vivo* reaction which is controlled by antigenic determinants encoded in or near the H-2 gene complex is not reflected in the *in vitro* cytotoxicity assays. In fact, several experimental approaches indicate that specific serum antibody present in responder strains of mice, directs NK cells in an antibody-dependent cytolytic and/or cytostatic reaction (ADCC) resulting in marrow graft rejection. This recent disclosure has clarified a mechanism for the recognition and rejection of allogeneic bone marrow which had remained obscure for many years and is futhermore the first *in vivo* demonstration of the long familiar *in vitro* ADCC reaction.

That some of the susceptible normal cell types (such as bone marrow stem cells and a subpopulation of thymocytes) are to be found in tissue sites where the local NK activity is minimal or non-existent, suggests that one of the functions of NK cells is to prevent trespass beyond normal physiological environments. Stem cell populations under an oncogenic influence undergoing expansion into 'forbidden clones' might thus be prime targets for NK cells once outside their normal tissue compartments. Since NK precursors may be found in both bone marrow and thymus and may be 'activated' by certain stimuli, NK cells may be expected to exert a local influence as well. This model might reasonably be extended to include the stem cells of tissues other than bone marrow, but has no implication for the importance of such a mechanism relative to others in controlling the emergence of neoplastic clones. The 'escape' from host restraint is a multifactorial process involving the breakdown of homeostatic mechanisms and evasion by failure of immunological recognition. Data derived from the study of murine and human leukaemias encourage the view that NK cells may have a role in the surveillance of lymphoproliferative malignancies but the extent of their involvement, and whether similar mechanisms are operative against tumours arising in other sites is debatable. The gap between theoretical prediction and experimental realisation thus remains.

Paterson Laboratories,
Christie Hospital and Holt Radium Institute,
Manchester M20 9BX,
UK.

M. Moore

**References**

BARLOZZARI, T., LEONHERDT, J., WILTROUT, R.H., HERBERMAN, R.B. & REYNOLDS, C.W. (1985). Direct evidence for the role of LGL in the inhibition of experimental tumour metastases. *J. Immunol.*, **134**, 2783.

BERAN, M., HANSSON, M. & KIESSLING, R. (1983). Human natural killer cells can inhibit clonogenic growth of fresh leukaemic cells. *Blood*, **61**, 596.

BOTTAZZI, B., INTRONA, M., ALLAVENA, P., VILLA, A. & MANTOVANI, A. (1985). In vitro migration of human large granular lymphocytes. *J. Immunol.*, **134**, 2316.

FODSTAD, O., HANSEN, C.T., CANNON, G.B., STATHAN, C.N., LICHTENSTEIN, G.R. & BOYD, M.R. (1984). Lack of correlation between natural killer activity and tumour growth control in nude mice with different immune defects. *Cancer Res.*, **44**, 4403.

GRIMM, E.A. & ROSENBERG, S.A. (1984). The human lymphokine-activated killer phenomenon. In *The Lymphokines,* Vol: 9, M. Landy & E. Pick (eds) Academic Press: London.

HOSHINO, T., KOREN, H.S. & UCHIDA, A. (eds.). (1984). Natural killer activity and its regulation ICS 641 Excerpta Medica, Amsterdam.

KARRE, K., LJUNGGREN, H.G., PIONTEK, G. & 4 others. (1985). Activation of cell mediated immunity by absence or deleted expression of normal cellular gene products, i.e. by "no self" rather than "non-self". *Immunobiol.*, **167**, 43 (Abstract).

KLEIN, E. (1983). Killing often spoken of as natural. Naming in clonal terms .... *Immunol. Today,* **4**, 97 (Annotation).

NEMLANDER, A., SAKSCHA, E. & HAYRY, P. (1983). Are 'natural killer' cells involved in allograft rejection? *Eur. J. Immunol.*, **13**, 348.

NEWLAND, A.C., CATOVSKY, D., LINCH, D. & 7 others. (1984). Chronic T cell lymphocytosis: a review of 21 cases. *Br. J. Haematol.*, **58**, 433.

ROBERTS, K. & MOORE, M. (1985). A clonal analysis of human peripheral blood lymphocytes displaying natural killer-like activity. *Eur. J. Immunol.*, (in press).

TARTOF, D., YUNG, C.W., CURRAN, J.J., LIVINGSTON, C. & THALJI, Z. (1984). Cells that mediate NK like cytotoxicity are present in the human delayed type hypersensitivity response. *Clin. Exp. Immunol.*, **58**, 462.

WARD, J.M., ARGILAN, F. & REYNOLDS, C.W. (1983). Immunoperoxidase localisation of large granular lymphocytes in normal tissues and lesions of athymic nude rats. *J. Immunol.*, **131**, 132.

WARNER, J.F. & DENNERT, G. (1982). Effects of a cloned cell line with NK activity on bone marrow transplants, tumour development and metastasis *in vivo*. *Nature,* **300**, 31.

WARNER, J.F. & DENNERT, G. (1985). Bone marrow graft rejection as a function of antibody-directed natural killer cells. *J. Exp. Med.*, **161**, 563.

YANAGI, Y., CACCIA, N., KRONENBURG, M, & 12 others. (1985). Gene rearrangement in cells with natural killer activity and expression of the $\beta$ chain of the T-cell antigen receptor. *Nature,* **314**, 631.